# Impact of zervimesine on the neuroinflammatory biomarker GFAP and related proteomic molecular correlates in plasma of participants from a phase 2 clinical trial in Alzheimer’s disease

**DOI:** 10.1186/s13195-026-02025-4

**Published:** 2026-04-06

**Authors:** Valentina Di Caro, Eunah Cho, Jill Thiel, Britney N. Lizama, Marleen J.A. Koel-Simmelink, Kiran Pandey, Duc Duong, Nicholas T. Seyfried, Michael Grundman, Charlotte E. Teunissen, Henrik Zetterberg, Kaj Blennow, Anthony O. Caggiano, Mary E. Hamby

**Affiliations:** 1 Cognition Therapeutics, 2403 Sidney Street Suite 261, Pittsburgh, 15203 PA USA; 2 Emtherapro Inc, Systems Biology, 1374 Pasadena Avenue NE, Atlanta, GA 30306 USA; 3 Emory University School of Medicine, Biochemistry, 1510 Clifton Road, 4001 Rollins Research Center, Atlanta, GA 30322 USA; 4 Global R&D Partners, LLC, 13236 Haxton Place, San Diego, CA 92130 USA; 5 Department of Neurosciences, University of California, 9375 Gilman Dr, San Diego, CA 92161 USA; 6 Department of Laboratory Medicine, VU University Medical Center, De Boelelaan 1117, Amsterdam, 1081 HV The Netherlands; 7 Department of Psychiatry and Neurochemistry, Institute of Neuroscience and Physiology, the Sahlgrenska Academy at the University of Gothenburg, Mölndal, Sweden; 8 Clinical Neurochemistry Laboratory, Sahlgrenska University Hospital, Mölndal, Sweden; 9 Department of Pathology and Laboratory Medicine, University of Wisconsin School of Medicine and Public Health, Madison, WI USA; 10 Wisconsin Alzheimer’s Disease Research Center, University of Wisconsin School of Medicine and Public Health, University of Wisconsin-Madison, Madison, WI USA; 11 Department of Neurodegenerative Disease, UCL Institute of Neurology, Queen Square, London, UK; 12 UK Dementia Research Institute at UCL, London, UK; 13 Hong Kong Center for Neurodegenerative Diseases, InnoHK, Hong Kong, China; 14 Centre for Brain Research, Indian Institute of Science, Bangalore, India

**Keywords:** Alzheimer’s disease, Zervimesine (CT1812), Glial fibrillary acidic protein (GFAP), Neuroinflammation, P-tau217, Plasma pharmacodynamic biomarkers, TMT-MS proteomics, Aβ oligomers, Investigational therapeutic, Phase 2 clinical trial

## Abstract

**Background:**

Zervimesine (CT1812) is an investigational brain-penetrant small molecule modulator of the sigma-2 receptor (S2R/*TMEM97*), currently in clinical development for the treatment of Alzheimer’s disease (AD) and dementia with Lewy bodies (DLB) that selectively prevents and displaces the binding of amyloid beta (Aβ) and α-synuclein oligomers from neuronal synapses. Given the mechanism of action, it was hypothesized that zervimesine might be more effective in patients with lower levels of AD pathology. Indeed, in the SHINE trial, a completed Phase 2, randomized, double-blind, clinical trial conducted in participants with AD, a robust, 95%, slowing of cognitive decline, as assessed via ADAS-Cog11, was observed in a pre-specified subgroup of participants with lesser AD pathology (i.e., low p-tau217 subgroup) compared to a 38% slowing in the overall modified intent-to-treat (mITT)) population.

**Methods:**

In SHINE, exploratory plasma biomarkers were assessed using both a targeted and an unbiased, proteomics discovery approach in plasma from participants at baseline and end of study. Treatment effects of zervimesine relative to placebo were assessed in both the mITT population and in a low p-tau217 subgroup, who entered the study with lower (< 1pg/ml) plasma p-tau217 concentrations. Plasma biomarkers Aβ40, Aβ42, GFAP, NfL and BD-tau were assessed using clinically validated targeted assays, along with untargeted TMT-mass spectrometry (MS)-based discovery proteomics followed by bioinformatic, pathway and correlation analyses.

**Results:**

Collectively, plasma biomarker findings in the low p-tau217 subgroup were more robust than in the mITT population. Levels of GFAP were significantly decreased and NfL, Aβ42 and Aβ40 levels trended towards a decrease with zervimesine compared to placebo. Proteomics analyses identified candidate pharmacodynamic biomarkers of zervimesine, and gene ontology and pathway analyses pointed to an impact on amyloid biology, trafficking, lipid metabolism, and immune response. Bioinformatics and correlation analyses with GFAP identified biomarkers that may reflect pathway engagement of S2R and/or decreased neuroinflammation.

**Conclusions:**

Exploratory plasma biomarker findings align with the degree of clinical benefit in SHINE mITT and low p-tau217 populations, and support future trial enrichment with patients with lower levels of pathology as defined by lower baseline plasma levels of p-tau217.

**Trial registration:**

July 20th, 2018 ClinicalTrials.gov Identifier NCT03507790 https://clinicaltrials.gov/study/NCT03507790.

**Supplementary Information:**

The online version contains supplementary material available at 10.1186/s13195-026-02025-4.

## Background

Zervimesine (CT1812) is an orally administered small molecule modulator of the sigma-2 receptor (S2R*/TMEM97*) in clinical development for treatment of Alzheimer’s disease (AD; NCT03507790; NCT05531656) and dementia with Lewy bodies (DLB; NCT05225415). The S2R (TMEM97) directly binds to the major component of the oligomer receptor, prion protein (PrP^C^) and preclinical studies have shown that zervimesine can displace toxic Aβ oligomers from binding to neuronal synapses. Treatment of neurons with zervimesine restores the functional and synaptic deficits incurred by Aβ oligomers, including the restoration of membrane trafficking, and the prevention of synapse loss [1]. Consistent with preservation of neuronal function and synaptoprotection, deficits in cognitive performance seen in transgenic mice with Aβ accumulation are normalized following zervimesine treatment [1]. Proof of zervimesine mechanism – displacement of toxic Aβ oligomers from binding neuronal synapses – was also demonstrated in mice, where after a single dose of zervimesine, levels of Aβ oligomers were increased in minutes to hours [1]. Following that, clinical proof of mechanism was seen in a small, single-dose clinical trial in AD patients where after a single dose of zervimesine, levels of Aβ oligomers rose over the 24 h period assessed [2]. Exploratory quantitative cerebrospinal fluid (CSF) proteomics data from two clinical trials in mild-to-moderate AD participants, SPARC (COG0105; NCT03493282 [3, 4]and an interim analysis from SHINE (SHINE-part A; COG0201; NCT03507790) [5, 6], support the impact of zervimesine on amyloid biology and synaptic function.

SHINE (COG0201; NCT03507790) is a completed Phase 2 randomized, double-blind, placebo-controlled clinical trial conducted in participants with mild to moderate AD [7, 8]. The primary objective of this study was to evaluate the safety and tolerability of zervimesine, with secondary objectives to evaluate CSF pharmacodynamic biomarkers, and key exploratory objectives to evaluate efficacy, data which was previously reported [7, 8].

A pre-specified subgroup analysis was also performed based off baseline levels plasma p-tau217. Substantial advancements in recent years has enabled the detection of newly developed and validated plasma biomarkers as a surrogate to indicate degree of AD pathology vs. the more expensive imaging tests or laborious CSF draws. Since plasma biomarker levels provide information on disease stage progression and potential for monitoring treatment effects, evaluation of baseline levels for specific plasma biomarkers can be a promising avenue to improve participant selection for clinical trials and assess treatment effects [9, 10].

The level of plasma p-tau217 is correlated with amyloid and tau pathology on PET imaging and has proven useful in identifying study participants for trials assessing the effects of anti-amyloid therapies [11–13]. A growing number of studies – with lecanemab and donanemab – are finding that the earlier stage of disease and/or the AD pathology, the greater the effect size in clinical benefit [14–17]. For example, reports of findings from the subgroup analysis of phase 3 trial TRAILBLAZER-ALZ2 suggest that donanemab is more effective in participants with less cognitive impairment and lower tau burden [16, 18]. Given this, and the mechanism of action of zervimesine in impacting features of AD that start at earlier disease stages and ensue – synapse loss and the present of toxic AβO [19], we hypothesized that a greater treatment effect might be observed in SHINE participants with lower p-tau217 levels within the range of p-tau217 levels in the mild-moderate AD population in SHINE. To test this hypothesis, a pre-specified subgroup analysis based off baseline plasma p-tau217 levels was also examined.

Participants were stratified by their baseline plasma p-tau217 level (above or equal to (i.e., the high p-tau217 subgroup), and below the median of 1pg/ml (i.e., the low p-tau217 subgroup), in addition to the overall modified intent-to-treat (mITT) analysis in mild to moderate AD participants. In mITT, participants treated with zervimesine experienced a slowing in cognitive decline (38% for ADAS-Cog11) and showed a decrease in CSF NfL levels relative to placebo (*p* = 0.016) [8, 20, 21]. SHINE participants treated with zervimesine who entered the study with lower plasma p-tau217 concentrations (below median baseline plasma p-tau217 of 1pg/mL (low p-tau217 group), experienced a more robust, compared to mITT, 95% slowing of cognitive decline, as measured by ADAS-Cog11, compared to placebo [22]. This robust clinical benefit represented a 2.7-point improvement on ADAS-Cog11 versus placebo over the 6 months study duration [8] and suggests that patients with lower levels of AD pathology (as marked via < 1 pg/ml p-tau217;) may have an improved response to therapy as compared to an Aβ^+^ mITT population not defined via p-tau217 levels.

An exploratory aim of SHINE was to analyze and identify plasma biomarkers to help elucidate the mechanism of action of zervimesine through S2R in AD patients, and to identify pharmacodynamic biomarkers of zervimesine efficacy in both the mITT population and the low p-tau217 subgroup. The p-tau217 subgroup analysis was performed to test the hypothesis that stratification of patients by the biomarker p-tau217 might identify a patient population likely to benefit from therapy.

The present manuscript describes for the first time the exploratory investigation of AD canonical plasma biomarkers such as Aβ40, Aβ42, GFAP, NfL and brain-derived tau (BD-Tau) using a targeted approach, as well as discovery proteomics, using TMT-mass spectrometry, to identify novel plasma pharmacodynamic biomarkers of zervimesine from the SHINE trial. Findings from the SHINE p-tau217 subgroup analysis identified a patient population most likely to respond to zervimesine treatment, with favorable findings of a biological impact – decrease in GFAP and trends in decreases in NfL and Aβ42 – consistent with improved clinical outcomes [7, 8]. This is an important take-away as this information may enable enrichment strategies for Phase 3, which might enhances the study’s ability to demonstrate efficacy by focusing on a more responsive, homogeneous population [9, 23]. Unbiased bioinformatic analyses of plasma proteomes identified zervimesine driven pharmacodynamic biomarkers/pathways and to identify potential molecular correlates to inflammatory processes/neuroinflammation occurring in AD pathology as assessed by GFAP levels in plasma. Identification of new biomarkers of target/pathway engagement arises from this study may help to better understand the biological impact of zervimesine in AD patients, and that may be considered to assess for replication of findings and monitor in future clinical studies.

Together, biomarker findings underline the biological impact of zervimesine in the SHINE trial on plasma biomarkers and biological processes related to AD. Data provide insights into the mechanism by which zervimesine may ameliorate AD through S2R, perhaps via dampening neuroinflammation in addition to slowing neurodegeneration, and identifies candidate pharmacodynamic biomarkers of zervimesine for use and further validation in future trials. The plasma biomarker findings reported herein that zervimesine can biologically impact the disease align with the favorable clinical outcomes of SHINE and provide further support for patient enrichment – using a baseline plasma p-tau217 cutoff – in future clinical trials, to enroll participants that may have a greater likelihood of responding favorably to treatment. Larger trials and follow-on studies are needed to further validate the findings of the present study.

## Methods

### Clinical trial design

SHINE (COG0201; NCT03507790) is a completed international, multi-center, randomized, double-blind, placebo-controlled parallel group 36-week Phase 2 clinical trial of zervimesine in adults with mild to moderate AD. Participants (*N* = 150), men and women aged 50–85 years with mild to moderate AD (MMSE 18–26), were screened for eligibility. AD diagnoses were confirmed by amyloid PET imaging or by CSF biomarkers measured at the screening visit. Participants were randomized 1:1:1 to receive placebo, or 100 or 300 mg zervimesine, orally, once daily for 6 months. Additional clinical trial details including results may be found at https://clinicaltrials.gov/study/NCT03507790 in addition to the topline manuscript [7, 8].

### mITT population and p-tau217 subgroup analyses

For the mITT population analyses, treatment effects were assessed for efficacy measures [8] and in the exploratory plasma biomarker and proteomics analyses. The mITT population included all randomized subjects who receive any amount of study drug and who have a baseline and at least one post-baseline assessment of the ADAS-Cog 11 total.

In the p-tau217 subgroup analyses, treatment effects were assessed for efficacy measures [8] and in the exploratory plasma biomarker and proteomics analyses in a pre-specified subgroup analysis. p-tau217 subgroups defined by a baseline p-tau217 plasma concentration below the median of 1.0 pg/ml, or at and above the median, referred to herein as the low and high p-tau217 subgroups, respectively. The low and high p-tau217 subgroups treatment populations were comparable at baseline (Supplementary Table 1). For this purpose of defining the subgroups, plasma p-tau217 was measured at baseline (AlzpathDx, Carlsbad, CA, USA) from SHINE trial participants and the median p-tau217 level was determined to be 1.0 pg/ml.

### Targeted canonical plasma biomarker assessments

All plasma biomarker analyses in this manuscript were exploratory and for the purpose of identifying pharmacodynamic changes of zervimesine. Only participants for whom timepoint-matched biofluids were available (participants for which plasma was collected at both baseline and 6 months (*N* = 117), were included in the analysis. Plasma levels of Aβ40, Aβ42, GFAP and NfL were measured using the “Digital immunoassay (SIMOA) Neuro4-Plex E Kit” (Quanterix, Billerica, MA, USA) and brain-derived tau using a SIMOA based assay developed in the Clinical Neurochemistry laboratory at Gothenburg University, Sweden [24]. For each plasma biomarker, change from baseline (CFB) was calculated (mITT, *N* = 117; low p-tau217 subgroup, *N* = 57; high p-tau217 subgroup, *N* = 60).

### Untargeted discovery TMT-MS proteomics assessments and bioinformatic analyses

TMT-MS was performed on the first 57 participants of SHINE (*N* = 57 participants, *n* = 20 placebo, *n* = 37 pooled zervimesine (100 mg and 300 mg), (Supplementary Fig. 1 A) with available plasma samples at both baseline and 6 months, who were also actively taking their treatment, as indicated by the bioanalysis of drug (zervimesine) exposure levels (i.e., “treatment-compliant” participants). In brief, plasma sample were processed via heparin affinity chromatography, followed by off-line fractionation and tandem mass tag mass spectrometry (TMT-MS) as previously described [5, 6, 25, 26]. Following this differential abundance analyses were performed, followed by pathway and correlation analyses (Supplemental Fig. 1B).

#### Differential abundance analyses

For differential abundance analyses, log2 change from baseline (CFB) abundances for each participant were first calculated. CFB treatment effects (zervimesine vs. placebo) were then assessed via analysis of variance (ANOVA) and significance assessed using an unadjusted p-value criterion *p* ≤ 0.05. Differential abundance analysis was assessed in the mITT population (*N* = 57 participants, *n* = 20 placebo, *n* = 37 zervimesine) as well as in the low p-tau217 subgroup (*N* = 24 participants; *n* = 8 placebo, *n* = 16 zervimesine) (Supplementary Fig. 1 A).

#### Pathway analyses

Biological function and relevance of differentially abundant proteins affected by zervimesine was assessed via Gene Ontology (GO) analysis, network associations via STRING (version 12.0), and functional processes and pathways via a curated database, MetaCore (versions 24.4.71900). For STRING, only UniProt identifiers with annotated protein names were input; protein-protein interaction (PPI) networks were exported, and pathway terms were interpreted. For MetaCore, both protein names and UniProt identifiers were used for analyses (Supplementary Fig. 1B).

#### Correlation analysis with plasma GFAP levels assessed using clinically validated, quantitative assay

**Fig. 1:**
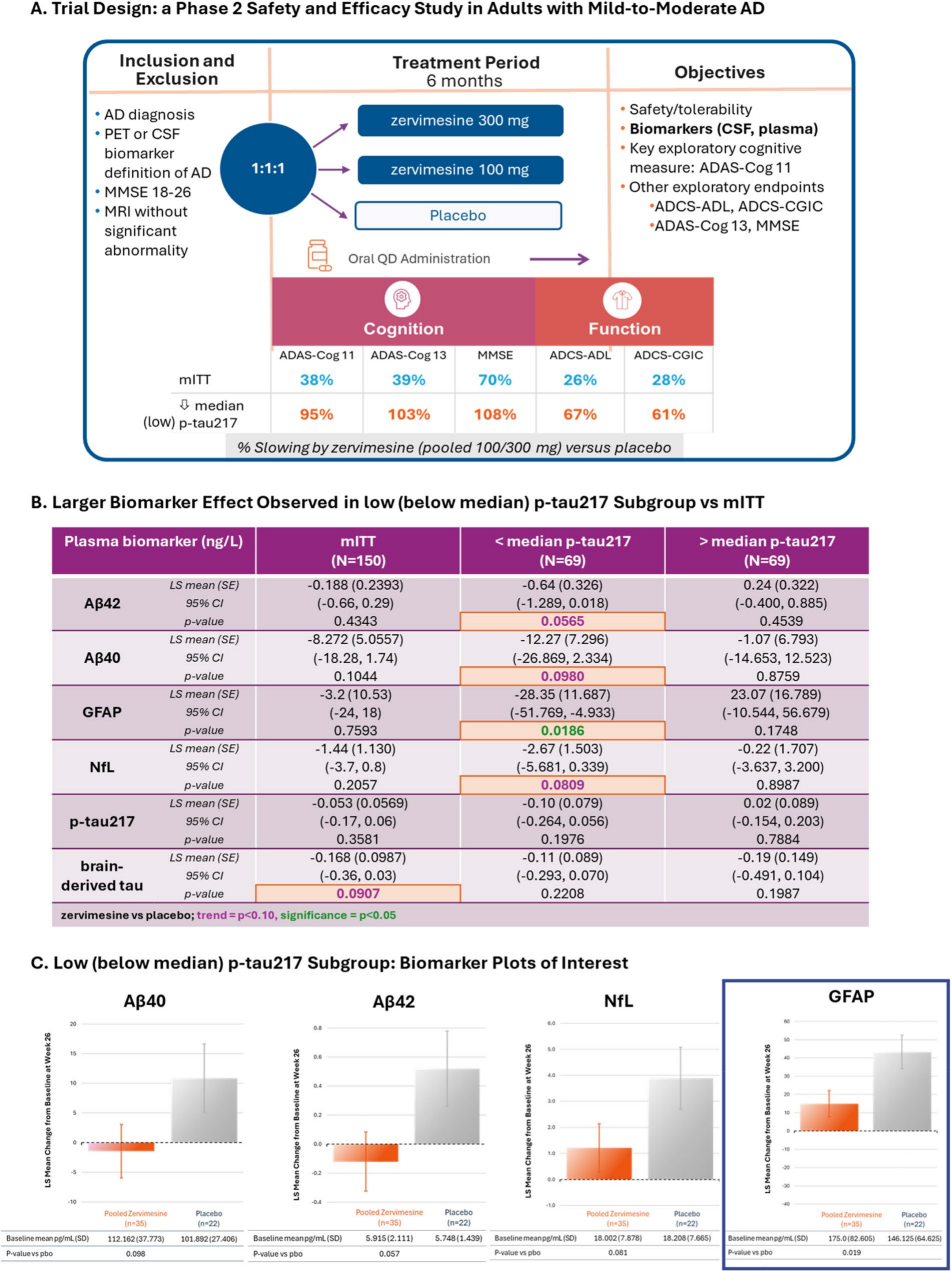
Clinical trial design, robust canonical biomarker effects in low p-tau217 subgroup. **A** SHINE clinical trial design, and previously reported summary of clinical and CSF biomarker outcomes. Patients treated with zervimesine showed a slower cognitive decline (% slowing shown) compared to placebo in mITT, an effect that was more pronounced in the low p-tau217 subgroup. **B** AD plasma biomarker findings listing the LS mean (SE) change from placebo, 95% confidence interval (CI), and significance after 6 months treatment with zervimesine in the SHINE trial participants. Statistical significance of a decrease in GFAP (p *≤* 0.05, bolded in green) and positive trends for decreases in Aβ42, Aβ40 and NfL (​p *≤* 0.10, bolded in purple) were seen in the low p-tau217 subgroup (*N* = 69; size of population included in the below median (low) p-tau217 subgroup). None of these differences were significant compared to placebo in the mITT (*N* = 150; size of participants included in the trial) and in the high p-tau217 subgroup (*N* = 69; size of population included in the above median (high) p-tau217 subgroup). **C** Representative graphs showing the robust effect sizes observed across biomarkers measured in below median (low) p-tau217 subgroup (*N* = 57; actual number of participants included in the analysis)

Pearson correlation was performed as a bivariate analysis to measure the strength of association between two variables (e.g., protein level and plasma GFAP levels detected by SIMOA) and the direction of the relationship. Given that plasma levels of the neuroinflammatory biomarker GFAP were found to be significantly reduced by zervimesine treatment (zervimesine vs. placebo) in the low p-tau217 subgroup (Fig. 1B, C), the subgroup which showed the greatest cognitive benefit, we rationalized that examination of proteins correlating with plasma GFAP levels may elucidate molecular (protein) and functional (pathway) mechanisms related to the underlying beneficial zervimesine treatment effect on dampening neuroinflammation in this cohort. To this end, in the low p-tau217 subgroup, Pearson correlation analysis was performed on CFB abundances of all proteins detected via TMT-MS against the CFB in levels of plasma GFAP, as measured by the quantitative SIMOA assay. A p-value criterion of *p* ≤ 0.05 was used to identify significantly associated correlates. Treatment effect and “Drug-selective” correlates were determined based on a statistical modelling previously described [26].

## Results

### Impact of zervimesine treatment on canonical plasma biomarkers using targeted assays

Plasma was collected at baseline and after 6 months of zervimesine treatment (Fig. 1A). Using clinically validated assays, levels of AD canonical biomarkers including Aβ42, Aβ40, Aβ42/Aβ40 ratio, GFAP, NfL, BD-Tau, and p-tau217 were measured and change from baseline (CFB) calculated for mITT population and the p-tau217 subgroups. In the mITT population, a trend (*p* = 0.09) towards a reduction of plasma brain derived (BD)-tau levels compared to placebo was observed, whereas no treatment-related changes were observed in CFB levels of plasma GFAP, NfL, Aβ42, Aβ40 (Fig. 1B and C), or Aβ42/40 ratio (zervimesine vs. placebo; LS mean 0.0019; SE 0.00148; *p* = 0.2048), following 6 months treatment with zervimesine. In the low p-tau217 subgroup, a statistically significant decrease in the neuroinflammatory marker GFAP (zervimesine vs. placebo; LS mean​ −28.35; SE 11.687; *p* = 0.0186) was observed along with trends (p *≤* 0.10) of reductions for Aβ42, Aβ40 and NfL in zervimesine vs. placebo (*N* = 57) (Fig. 1B and C). Notably, despite trends seen towards a lowering of Aβ42 and Aβ40, no change in Aβ42/40 ratio (zervimesine vs. placebo; LS mean 0.00; SE 0.001; *p* = 0.5401) was observed. In contrast to the low p-tau217 subgroup, no treatment effects or trends were observed in any biomarkers assessed in the high p-tau217 subgroup (*N* = 60) (Fig. 1B).

### Proteomics discovery of pharmacodynamic plasma biomarkers of zervimesine

**Fig. 2:**
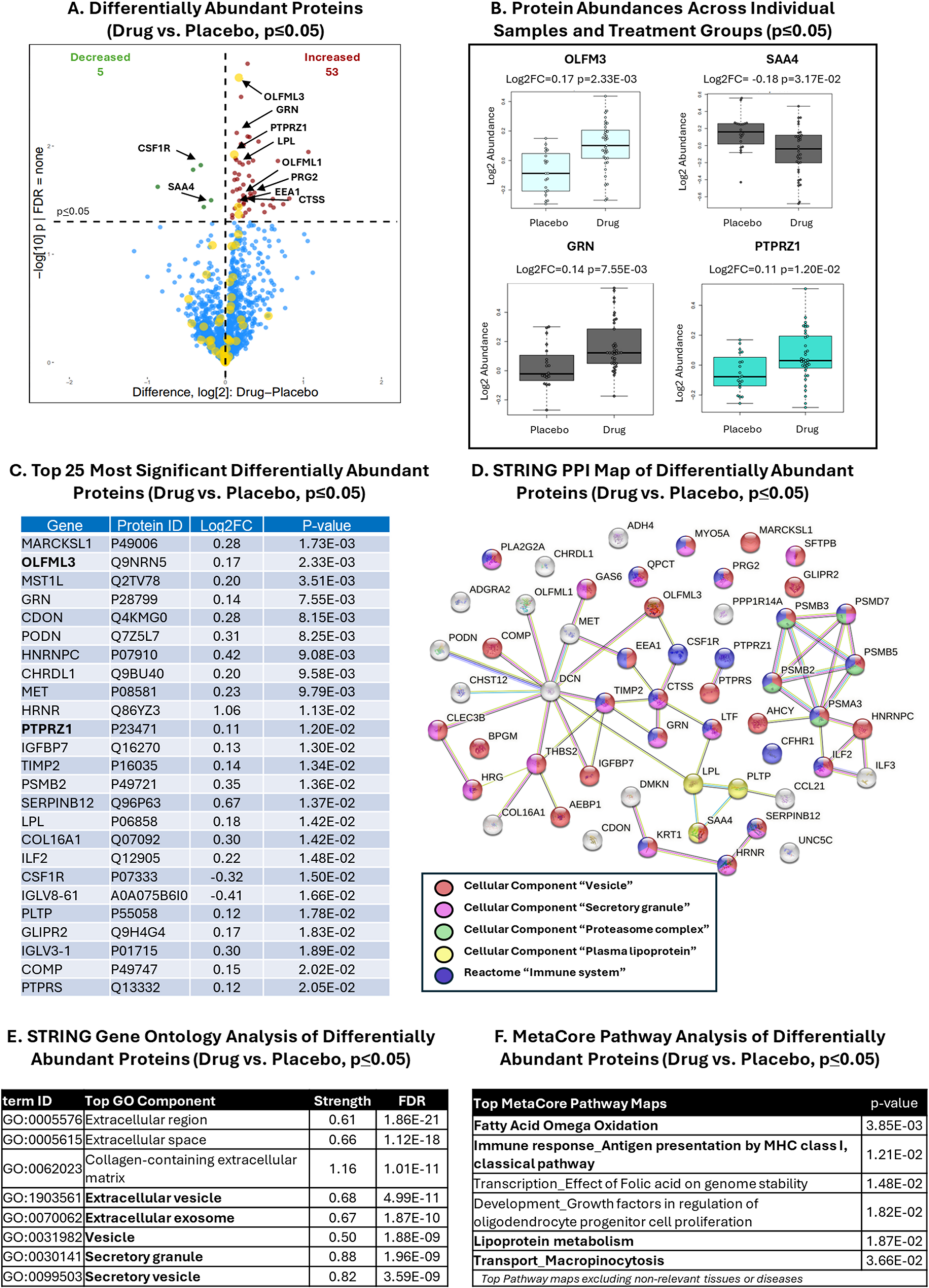
Analysis of differentially abundant proteins in zervimesine vs. placebo-treated patient plasma proteomes in mITT population.** A** Abundances of proteins measured using TMT-MS were compared between zervimesine and placebo-treated patient plasma after 6 months. Proteins that were significantly differentially abundant in zervimesine vs. placebo plasma proteomes are shown on the volcano plot to visualize directionality (58 proteins met significance criterion of *p* ≤ 0.05 for zervimesine vs. placebo; 5 decreased (upper left quadrant, green dots), 53 increased, (upper right quadrant, red dots)). Each colored dot corresponds to a protein, larger yellow circles indicate “AD Priority biomarkers”. Proteins of interest are indicated with black arrows. **B** Example box plots of proteins of interest demonstrate the log2 abundance and variance across individuals. Zervimesine vs. placebo log2 fold-change (FC) and p-values as indicated. **C** Top 25 most significant differential abundant proteins (zervimesine vs. placebo, *p* ≤ 0.05); in bold, “AD Priority biomarkers”. **D** STRING PPI map of the significantly (*p* ≤ 0.05) differentially abundant proteins (medium confidence threshold 0.4; PPI enrichment p-value = 6.31e-11*)*. Colored nodes refer to GO Cellular Component as indicated in key. **E**-**F** Pathway analyses via STRING (sorted by FDR) (**E**) and MetaCore (sorted by p-value) (**F**) of differentially abundant proteins in plasma proteome show vesicle, transport, immune response and lipoprotein metabolism to be most significantly altered pathways

Across mITT participant plasma samples, more than 2000 proteins were detected via TMT-MS proteomics, of which 58 proteins were significantly differentially abundant with zervimesine treatment (Fig. 2A: zervimesine vs. placebo *p* ≤ 0.05, 53 increased (red), 5 decreased (green)). Proteins relating to AD, S2R biology, or downstream effects of the zervimesine mechanism of action were identified (Fig. 2A, black arrows). Proteins shown in volcano plot are: CSF1R, colony stimulating factor 1 receptor; CTSS, cathepsin S; EEA1, early endosome antigen 1; GRN, progranulin; LPL, lipoprotein lipase; OLFML1, olfactomedin-like 1; OLFML3, olfactomedin-like 3; PRG2, proteoglycan 2, pro eosinophil major basic protein; PTPRZ1, protein tyrosine phosphatase receptor type Z1; SAA4, serum amyloid A4. Priority AD biomarkers (i.e., biomarkers disrupted in or genetically linked to AD (24–26)) are indicated in yellow (Fig. 2A), with significant AD priority biomarkers listed (Supplementary Table 2). Box plots of proteins of interest are shown to demonstrate zervimesine treatment effects and the variance across individuals (Fig. 2B).

To understand the biological pathways and the protein functions affected by zervimesine, STRING pathway analysis of the differentially abundant proteins was performed (Fig. 2D, E). A protein-protein interaction (PPI) map displays the interconnectivity of proteins and reveals a prominent hub cluster of proteasomes (green), vesicle (red), plasma lipoprotein (yellow), secretory granule (pink) and immune system (purple) related networks (Fig. 2D). Top ranked gene ontology (GO) Component terms included several terms related to vesicles including “extracellular vesicle”, “extracellular exosomes”, “vesicle”, “secretory granule” and “secretory vesicle” (Fig. 2E). MetaCore pathway analysis, which uses a distinct, curated database of predesignated pathways, identified impacted pathways related to known S2R functions and/or AD that included “Fatty Acid Omega Oxidation”, “Immune Response-Antigen presentation by MHC class I, classical pathway”, “Lipoprotein metabolism” and “Transport-Macropinocytosis” (Fig. 2F).

**Fig. 3:**
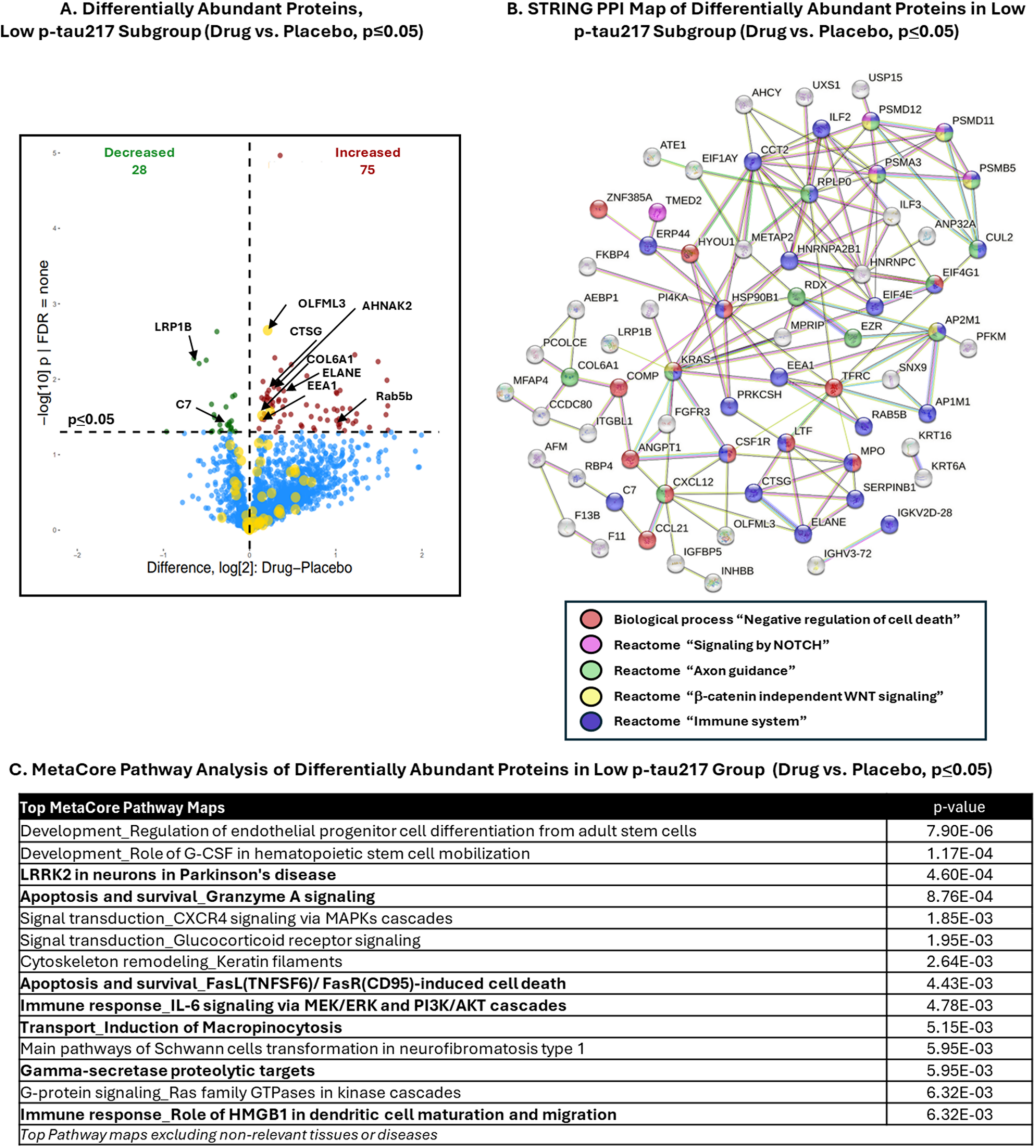
Analysis of differentially abundant proteins in zervimesine- and placebo-treated patient plasma proteomes in the low p-tau217 subgroup. **A** Abundances of proteins measured using TMT-MS were compared between zervimesine-treated and placebo-treated patient plasma after 6 months in the low p-tau217 subgroup. Proteins that were significantly differentially abundant in zervimesine vs. placebo plasma proteomes are shown on the volcano plot to visualize directionality (103 proteins met significance of *p* ≤ 0.05 for zervimesine vs. placebo, in the low p-tau217 group; 28 decreased (upper left quadrant, green dots), 75 increased, (upper right quadrant, red dots)). Each colored dot corresponds to a protein, larger yellow circles indicate “AD Priority biomarkers”; proteins of interest are indicated with black arrows. **B** STRING PPI map of the significantly (*p* ≤ 0.05) differentially abundant proteins in the low p-tau217 subgroup (medium confidence threshold 0.4; unconnected proteins not shown; PPI enrichment p-value = 1.41e^− 12^*)*. Colored nodes refer to GO Biological Process Terms as indicated in key. **C** Pathway analyses using MetaCore (sorted by *p*-value) of differentially abundant proteins at *p* ≤ 0.05 in plasma proteome in the low p-tau217 group show transport, immune response and apoptosis and survival to be most significantly altered pathways

A proteomic analysis was also performed in the low p-tau217 subgroup of participants, given that the most robust treatment effects in cognitive and functional outcomes (Fig. 1A), and canonical plasma biomarkers (Fig. 1B, C), were observed in the low p-tau217 subgroup. Differential abundance analysis identified 103 statistically differentially abundant proteins with zervimesine treatment (Fig. 3A: zervimesine vs. placebo *p* ≤ 0.05, 75 increased (red), 28 decreased (green)). Proteins shown in volcano plot: C7, complement 7; COL6A1, collagen type V1 alpha 1 chain; CTSG, cathepsin G; EEA1, early endosome antigen 1; LRP1B, LDL receptor related protein 1B; OLFML3, olfactomedin like 3; Rab5b, member RAS oncogene family; AHNAK2, AHNAK nucleoprotein 2; ELANE, elastase, neutrophil expressed. Pathway analysis, using STRING and MetaCore, of the zervimesine-impacted proteins (P *≤* 0.05) illuminated apoptosis and survival, axon guidance and, similar to the mITT population, transport and immune response as top pathways enriched (Fig. 3B and C, and Supplementary Table 2).

### Zervimesine-impacted plasma proteins correlated with plasma GFAP levels

**Fig. 4:**
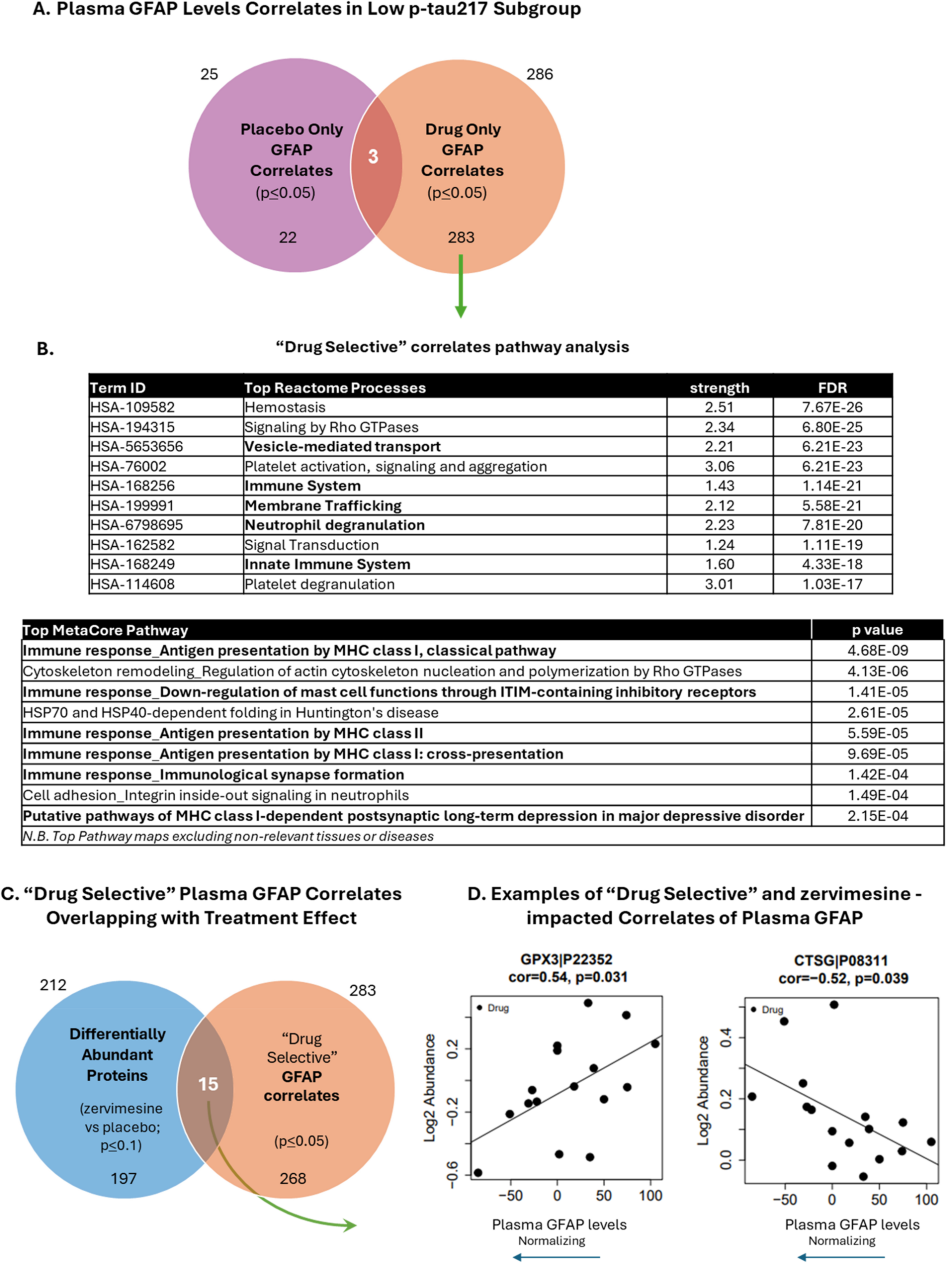
Proteomic correlates of plasma GFAP in low p-tau217 subgroup. **A** Proteomic change from baseline (CFB) abundances correlated with CFB plasma GFAP levels were identified in the low p-tau217 subgroup based off treatment, Placebo treated “Placebo only” (purple circle, 25 total correlates) and drug treated “Drug only” (orange circle, 286 correlates), via Pearson correlation analysis. Correlates identified irrespective of treatment (dark orange overlap, 3 correlates) were removed from “Drug only” plasma GFAP correlates to facilitate analysis of correlates that were treatment (drug)-selective (orange semicircle, 283 correlates). **B** The 283 “Drug Selective” protein correlates were analyzed using STRING and MetaCore. Tables display top-ranked Reactome terms identified by STRING (top panel; sorted by FDR) and MetaCore pathways (bottom panel; sorted by p-value). Proteins associated with STRING included RAB10, RAB14, RAB7A for “Vesicle-mediated transport” and HSP90AA1, ITGB1, LAMP1 and IGF2R for “Immune System”. **C** Of the 283 “Drug Selective” proteins identified that are correlated with plasma GFAP levels, 15 were determined to also be differentially abundant (zervimesine vs. placebo, *p* ≤ 0.10 in the low p-tau217 subgroup). **D** Scatter plots of two representative proteins of the 15, GPX3 and CTSG, show favorable correlation of GPX3 and CTSG CFB log2 abundance levels with plasma CFB GFAP levels across individual zervimesine-treated patients

Elevated levels of GFAP in plasma and serum are associated with the degree and rate of neurodegeneration, Aβ positivity and cognitive decline in individuals with AD [27, 28]. Given that a decrease in plasma GFAP levels was observed with zervimesine treatment compared to placebo in the low p-tau217 subgroup Pearson correlation analysis was performed on CFB GFAP levels, measured by a clinically validated assay, and plasma protein abundances of all TMT-MS proteins in the low p-tau217 subgroup (*N* = 16). In participants treated with zervimesine, a total of 286 TMT-MS-detected plasma proteins were found to be significantly (*p* ≤ 0.05) correlated with plasma GFAP levels (Fig. 4A, full orange circle). Of these, three proteins (dark orange intersection of two circles) were also found to be significantly (*p* ≤ 0.05) correlated with plasma GFAP levels in the placebo group. To focus on identifying molecular correlates of plasma GFAP levels that were selectively impacted by zervimesine treatment, only the 283 “Drug Selective” protein correlates (r≥|0.50|) (orange semicircle; top correlates shown in Supplemental Table 3), were selected for pathway analyses (Fig. 4B). The top-ranked STRING “Reactome” GO terms included “Vesicle-mediated transport,” “Membrane trafficking” and “Immune System” (Fig. 4B; top). MetaCore analysis of the 283 correlates indicated multiple pathways related to immune response amongst the top pathways impacted by zervimesine (Fig. 4B; bottom).

To identify which of the 283 “Drug Selective” protein correlates with plasma GFAP levels (Fig. 4C, full orange circle) were significantly impacted or trending towards a change in abundance, comparative analysis was performed with 197 differentially abundant proteins (zervimesine vs. placebo, *p* ≤ 0.10, in the low p-tau217 subgroup; full blue circle). Fifteen proteins (Fig. 4C, brown overlap) met both correlation and differential abundance criteria. Scatter plots of two of these proteins – glutathione peroxidase 3 (GPX3) and cathepsin G (CTSG) – are shown, demonstrating examples of a positive and negative correlation of change in abundance with improvement in plasma GFAP levels (Fig. 4D) (i.e., normalization of plasma GFAP levels towards healthy control levels) [29].

**Fig. 5:**
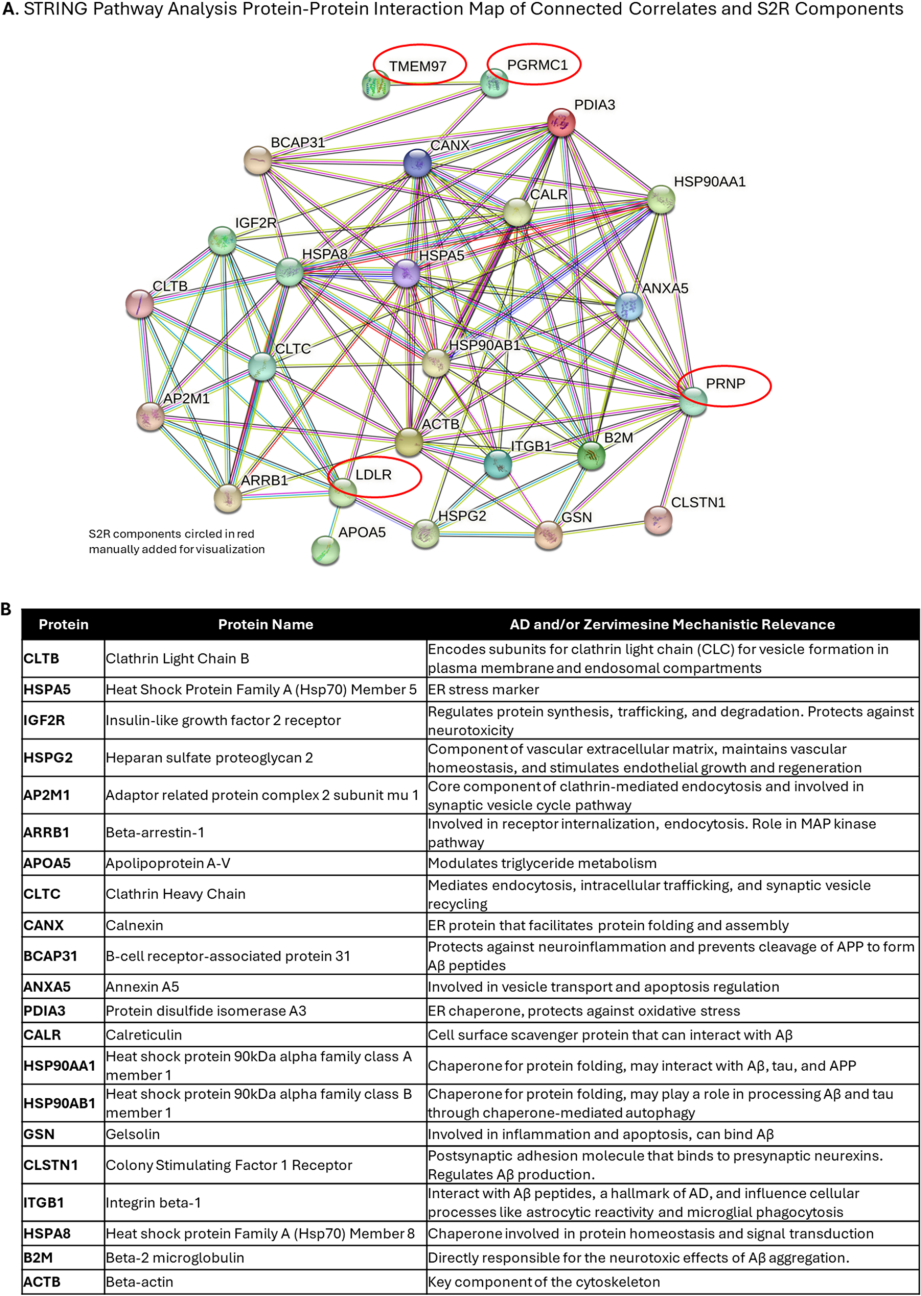
“Drug selective” correlates of plasma GFAP levels in the low p-tau217 subgroup relate to S2R biology. S2R complex components (indicated by red ovals) were manually added to the list of 283 “drug selective” correlates for STRING protein-protein interaction (PPI) mapping. **A** PPI map shows only the “Drug Selective” correlates that were found to be interact with S2R components (medium confidence threshold 0.4). **B** Table of the 21 correlates with plasma GFAP levels found to interact with the S2R complex components and their known biological functions relevant to AD or the zervimesine mechanism of action through S2R

To identify candidate pharmacodynamic biomarkers of zervimesine that may reflect target/pathway engagement, and for mechanistic hypothesis generation, the S2R complex components (indicated by red ovals in Fig. 5A) were manually added to the STRING PPI map, illustrating the relationship of “Drug Selective” correlates with zervimesine mechanism of action through S2R (Fig. 5). The functions of these 21 proteins are briefly summarized (Fig. 5B) and are related to AD biological pathways, zervimesine mechanism of action, or both.

**Fig. 6:**
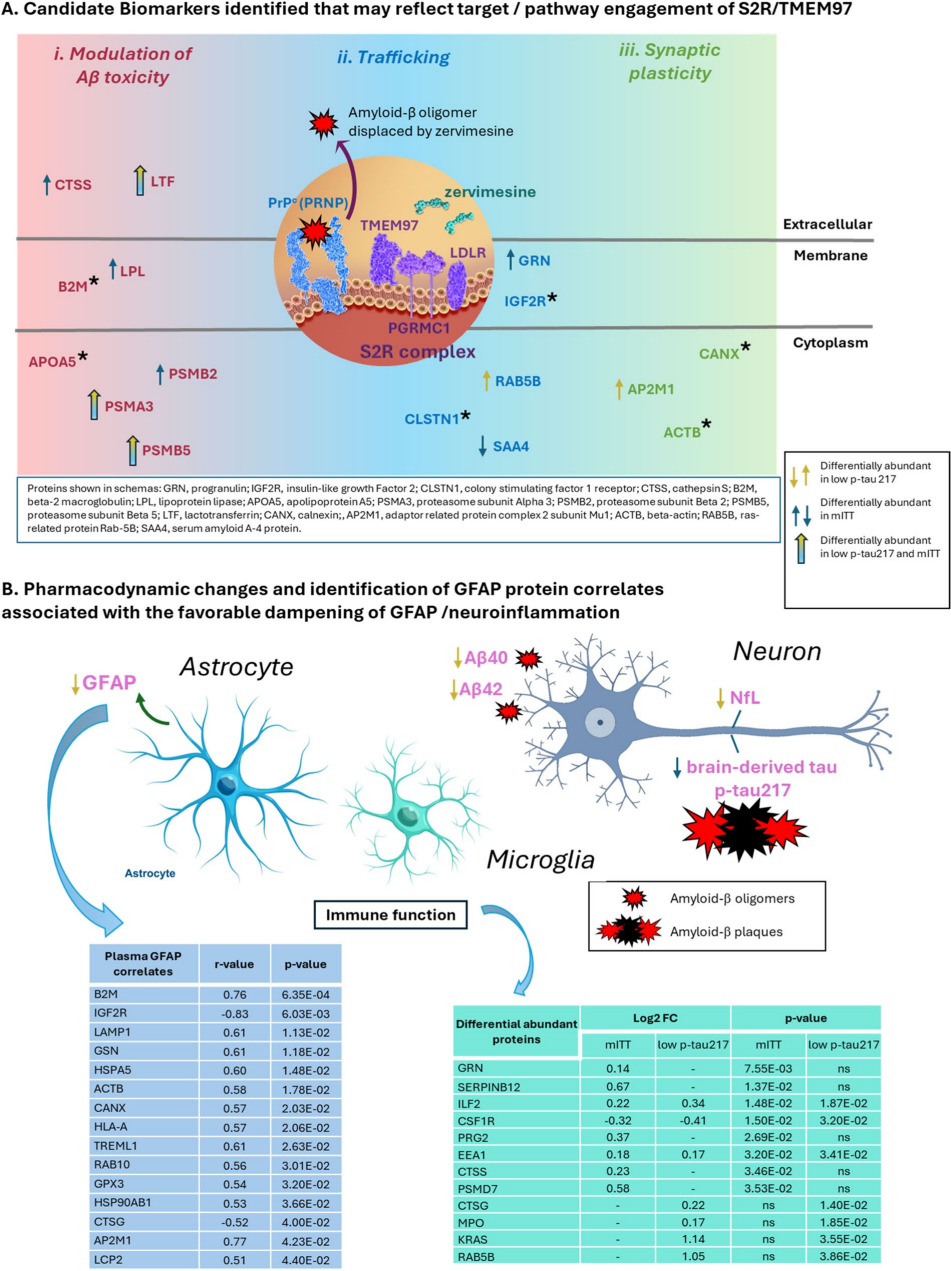
Summary of candidate biomarkers of zervimesine related to AD and neuroinflammation. **A**. Schema to summarize candidate biomarkers of pathway/target engagement related to zervimesine mechanism of action through S2R. Zervimesine (light green) binds S2R/TMEM97 (purple), which is localized on synapses in a complex with the oligomer receptor (prion protein; PRNP) the LDL receptor (LDLR) and PGRMC1. Binding of zervimesine to S2R induces a conformational change in the oligomer receptor complex (blue), disengaging the Aβ oligomers (red) from the synapse. Dark yellow and blue allows indicate directionality for differentially abundant proteins in the low p-tau217 subgroup and mITT population, respectively; two-color arrow indicates differential abundant proteins in both groups, “*”=protein is correlated with GFAP plasma levels. **B**. Canonical biomarkers (pink font) found to be trending (NfL, Aβ40, Aβ42, brain-derived tau) or affected (GFAP) with zervimesine treatment using targeted assays in either the mITT (blue arrow) or low p-tau217 group (yellow arrow; all decreased) (Fig. 1B schema) in relation to cell type and amyloid pathology (Aβ oligomers and plaques). In the left table, biomarker correlates with GFAP plasma levels in low p-tau217 subgroup (*p* ≤ 0.05) (left table) and correlates that are differentially abundant (*p* ≤ 0.05), sorted by p-value, along with pharmacodynamic biomarkers related to immune function identified in the differential abundance analyses in the mITT population and/or p-tau217 subgroup (right table; “ns”= nonsignificant; thus, “-” no Log2FC reported

Candidate biomarkers from the TMT-MS study that are linked to zervimesine mechanism of action are summarized (Fig. 6A). Several biomarkers identified in either or both the mITT population and p-tau217 subgroup analyses, supports that zervimesine modulates i) Aβ toxicity (CTSS, B2M, LPL, APOA5, PSMA3, PSMB2, PSMAB5, LTF), (ii) neuronal trafficking (GRN, IGF2R, CLSTN1, RAB5B, SAA4), and (iii) synaptic plasticity (CANX, AP2M1, ACTB). Additionally, the key canonical biomarker findings and the proteomic correlates of plasma GFAP levels may provide mechanistic insights and prompt further hypothesis generation on how zervimesine may impact neuroinflammation (Fig. 6B).

## Discussion

The exploratory plasma biomarker analysis from the SHINE clinical trial demonstrated that zervimesine can modulate, over a 6 month treatment period, levels of plasma canonical biomarkers associated with AD pathology and disease progression in a low p-tau217-defined population, effects which were muted or lost in the mITT population plasma biomarker analysis. In conjunction with larger, more favorable clinical outcomes in the low p-tau217 population, findings are in support that mild to moderate AD patients with low (< 1 pg/ml) p-tau217, may have a better response to treatment with zervimesine. Exploratory proteomics analyses helped to identify candidate biomarkers that may be tracked in future trials, and shed light on possible mechanistic, biological underpinnings of the favorable effects observed with zervimesine in SHINE. Future studies will be warranted to better delineate how, and to what extent, zervimesine may impact neurodegeneration, neuroinflammation and amyloid biology.

Given the zervimesine mechanism of action, and the growing evidence that Aβ targeted therapies may be more effective in participants that are earlier stage or have less brain pathology [17, 18, 30, 31], we hypothesized that a larger treatment effect would be observed in participants with lower levels of pathology than those with higher levels. This hypothesis was pre-specified in the statistical analysis plan for the SHINE trial using an indirect measure of brain pathology by assessing baseline plasma p-tau217 levels and using the median value to generate “low” and “high” p-tau217 subgroups. p-tau217 was selected for this substudy as it is an extensively validated to be an excellent indicator of degree of amyloid as assessed via CSF analysis and amyloid PET, and tau levels as assessed via tau PET [9, 13, 14, 32], and for practical reasons, as if the hypothesis was proven correct, this might enable use of this plasma biomarker to enrich for a responder population in Phase 3, vs. the more expensive imaging tests or laborious CSF draws. Further, the ability to recruit patients based off a plasma biomarker test is a huge advantage, for many reasons: Plasma biomarkers are less invasive, less costly, and more time-efficient measurements than CSF or neuroimaging biomarkers [9].

Results for the plasma biomarker analysis for the SHINE trial in the low p-tau217 subgroup aligned with our clinical observations towards improved cognition and functioning. This finding is important as it extends our understanding on defining the limits of potential mechanisms targeted by a given therapeutic that might be more effective in exerting therapeutic benefit in patients with either earlier, vs. later, stages of AD, or in patients with less vs. more AD pathology; in this case as defined by p-tau217. While other studies (e.g., TRAILBLAZER) assessed subgroups based off tau PET – indicating that individuals with lower AD pathology have greater response to amyloid-based therapies (e.g. Donanemab TRAILBLAZER 2) [18], here we show that using a plasma biomarker that reflects degree of AD pathology (both amyloid and tau) may be sufficient to identify a responder population, which may facilitate patient enrichment for future Phase 3 trials. This underlines the importance and relevance of patient selection based on pathological stage of disease, and perhaps suggests targeting patients with less pathology for trial enrollment may be of benefit to assess therapeutics targeting Aβ directly (Aβ lowering with Aβ monoclonal antibodies), or indirectly (by targeting Aβ oligomer-mediated toxicity) by modulating S2R.

Indeed, in a subgroup analysis defined by the median of the baseline plasma p-tau217 concentrations, the low p-tau217 subgroup in which participants had levels below the median value of 1pg/ml, the decrease of plasma GFAP levels observed was statistically significant (p *≤* 0.05). Increased GFAP, associated with astrogliosis, is a hallmark of and linked to pathological processes in Alzheimer’s disease, particularly amyloid build-up, and neuroinflammation [27]. Elevated secreted GFAP levels is a well-established marker of astrogliosis, detectable in plasma due to blood-brain-barrier disruption, and elevated in plasma of patients with AD. Moreover, plasma GFAP is a sensitive biomarker for detecting reactive astrogliosis, a hallmark of neuroinflammation, as well as Aβ pathology, even in early stages of AD [33]. Reduction of plasma GFAP in zervimesine-treated participants compared to control is consistent with the potential effects of zervimesine in modulating inflammatory or immune response pathways, as reported in prior exploratory CSF biomarker studies [5, 6], although the precise molecular mechanisms underlying the lowering of GFAP and the impact on neuroinflammation remains elusive. Proteomics analyses performed herein may be helpful in providing some good hypotheses to follow-up on in future studies.

Nominally significant trends (p *≤* 0.10) in reducing plasma levels of Aβ40, Aβ42, and NfL were also observed in zervimesine vs. placebo treated participants, which provides support for a role of zervimesine in impacting amyloid biology, neuroinflammation and neurodegeneration in patients with mild to moderate AD that have lower than 1 pg/ml p-tau17 levels at baseline using the AlzPath Dx assay. Notably, the trends seen of decreases in Aβ40, Aβ42 and NfL align with that observed in CSF of zervimesine treated participants compared to placebo (NfL *p* = 0.016, Aβ40 *p* = 0.305, and Aβ42 *p* = 0.150 in mITT population) [8]. The Aβ42/40 ratio is decreased in AD patients reflecting increased brain amyloid plaque load, and has been helpful in the diagnosis of AD [34, 35]. Zervimesine, showed no impact on Aβ42/40 ratio in either the mITT or low p-tau217 populations suggestive of no impact on total amyloid brain plaque levels. Thus, the reduction of Aβ40 and Aβ42 levels in zervimesine-treated participants compared to placebo may point to downstream effects of zervimesine on Aβ dynamics, particularly given zervimesine mechanism of action in engaging S2R and displacing Aβ oligomers from neuronal synapses [1]. More specifically, the oligomer receptor prion protein, PrP^C^, not only binds TMEM97 (S2R) [36], it is also known to bind amyloid precursor protein (APP) [37], from which Aβ40 and Aβ42 is derived. Given that PrP^C^ can bind APP and can alter levels of Aβ40 and Aβ42 [38], it is interesting to speculate that perhaps zervimesine treatment through S2R’s interaction with PrP^C^ may somehow impact this process. Future studies would be helpful to better understand the direct molecular mechanisms underlying this finding and understand the possible relationship to clinical effect. Decrease of CSF NfL levels in the SHINE zervimesine-treated participants further underlines the role of zervimesine in slowing neurodegeneration. NfL is a recognized biomarker of neurodegeneration for multiple neurodegenerative disorders, including AD, in which CSF levels are elevated relative to healthy non-demented control. Reduction in NfL levels in zervimesine-treated participants compared to placebo is consistent with preclinical studies demonstrating zervimesine rescue of synaptic loss and neuronal function in vitro and preservation of cognitive function in an in vivo AD model [1].

Previously, only CSF proteomics analyses have been reported to assess the biological impact of zervimesine in AD participants [5, 6, 26]. These exploratory analyses supported an impact of zervimesine on AD pathophysiology [1, 36, 39, 40] including amyloid biology, synaptic function [4–6], inflammation and lipoprotein-related biological processes [5, 6, 41], in agreement with preclinical studies [36, 39]. This is the first report of an exploratory discovery proteomics analysis in plasma following 6 months zervimesine treatment in participants with mild to moderate AD. Quantitative proteomics in the mITT population and the low p-tau217 subgroup identified plasma biomarkers that were significantly altered with zervimesine in the SHINE trial. Among these proteins were many associated with AD pathology, proteins possibly suggesting pathway/target engagement, and those providing early proof of zervimesine’s mechanism of action though S2R. Pathway analysis, using STRING and Metacore, of the significantly differentially abundant proteins (zervimesine vs. placebo) in both the mITT population and the low p-tau217 subgroup, determined that the impacted proteins are mainly associated to pathways related to transport, lipoprotein metabolism biology, cell survival and immune response. While findings require replication in larger cohorts treated with zervimesine (in future trials), many of the individual proteins found to be differentially abundant in zervimesine treated participants compared to placebo are related to zervimesine’s mechanism of action. It follows that these proteins may be potential biomarkers of target/pathway engagement of zervimesine. These candidate biomarkers include early endosome antigen 1 (EEA1), identified in mITT and low p-tau217 groups, and progranulin (GRN) identified in the mITT population. EEA1 is a key protein is a key marker of early endosomes that plays a crucial role in the endocytic trafficking and amyloidogenic processing of amyloid precursor protein (APP) [42], and altered expression of endosomal proteins including EEA1, can contribute to the AD progression, particularly in relation to Aβ accumulation [43]. Progranulin (GRN) regulates lysosomal biogenesis, inflammation and stress response [44]. GRN has been genetically linked to the neurodegeneration, with loss of function mutations or decreased levels have increased risk for neurodegenerative diseases including AD [44]. Zervimesine increased GRN levels in this study, in the mITT proteomics analysis; which is worthy of mention given that enhancing GRN levels is a therapeutic strategy being pursued in drug development for neurodegenerative conditions [44].

Proteoglycan 2, pro eosinophil major basic protein (PRG2), a protein involved in axonal growth and vesicle biology [45], is a pharmacodynamic biomarker previously identified in a CSF proteomic analysis of a 29-day study conducted with zervimesine (SEQUEL; NCT04735536) found to be correlated with drug level and directly linked to S2R/*TMEM97* via low-density lipoprotein receptor (LDLR), which is known to bind directly to TMEM97 [46]. Here we find PRG2 to be significantly differentially abundant with zervimesine compared to placebo. While intriguing, further studies are necessary to understand the relevance of this finding.

Lipid metabolism, a process dysregulated in AD, and proteins including lipoprotein lipase (LPL), serum amyloid A-4 (SAA4) and low-density lipoprotein receptor-related protein 1B (LRP1B), were also biomarkers impacted by zervimesine treatment in the mITT or low p-tau217 populations. Of note, LPL is a key enzyme involved in lipid metabolism. By interacting with lipoprotein receptors, such as the direct interactor with S2R (TMEM97), LDLR [46], LPL promotes the internalization of lipoproteins, contributing to lipid homeostasis and receptor-mediated signaling in neurons. In addition, LPL maintains the balance of membrane composition and fluidity, which is crucial for proper synaptic function and plasticity and is necessary for effective neurotransmitter release [47].

As previously discussed (above), the present study shows that levels of plasma GFAP in a subgroup of participants, who entered the study with lower plasma p-tau217 concentrations (low p-tau217 subgroup), were significantly lower in zervimesine, compared to placebo, treated participants. Given that GFAP is a marker of reactive astrogliosis, a hallmark of neuroinflammation, and given that plasma GFAP plasma levels correlations with Aβ pathology and disease progression [27] we sought to perform an exploratory analysis to seek to understand what proteins and biological pathways were associated with this change, by leveraging our TMT-MS-detected proteomics dataset. In zervimesine-treated participants, specifically in the low p-tau217 subgroup, GFAP CFB levels were significantly correlated with CFB abundances of 283 “Drug Selective” proteins. Given this analysis was performed using an unadjusted p-value of p *≤* 0.05, it is likely that 5% of the proteins may be false-positives. Thus, additional filters were applied to understand which of these proteins were known to associate with S2R, and pathway analysis was performed. We reasoned that proteins that might be false-positives would have a decreased likelihood of being connected to S2R, by random chance, and used pathway analysis as well, as it is well-understood that proteins altered by random chance (not driven by the biology, or treatment in this case), would not be expected to be enriched into a given pathway by random chance [48]. Similar to the STRING and Metacore analysis performed with the set of identified zervimesine pharmacodynamic biomarkers (differentially abundant proteins *p* ≤ 0.05), pathway analyses of the plasma GFAP level correlates identified GO terms like “Vesicle-mediated transport”, “Membrane Trafficking” and immune system related pathways, pathways that were corrected for multiple comparisons (FDR *≤* 0.05), reducing the likelihood that a given pathway was noise/a false positive [48]. Of the 283 “Drug Selective” proteins correlated with the plasma GFAP levels (P *≤* 0.05), 15 were also impacted by zervimesine treatment (zervimesine vs. placebo, *p* < 0.05). Glutathione peroxidase 3 (GPX3) and cathepsin G (CTSG), were of particular interest given the favorable directionality in plasma GFAP levels towards disease normalization. GPX3 is an enzyme involved in the antioxidant system and its expression is altered in AD [49]. CTSG, was also found differentially expressed in the low p-tau217 subgroup and is involved in Aβ deposition and like CTSS can modulate immune responses [50].

Other proteins correlated with plasma GFAP levels and directly linked to S2R components (Fig. 5A) include CLSTN1, IGF2R, HSPA5, HSP90AB1, and the apolipoprotein APO5. CLSTN1 (calsyntenin-1) and IGF2R (the insulin-like growth factor 2 receptor) are both proteins associated with AD. IGF2R is important for memory, and its dysregulation may contribute to AD pathology. IGF2R levels decrease with the presence of the *APOE4* allele, which is associated with increased risk for AD and overexpression of IGF2R is also associated to increase Aβ production [51, 52]. CLSTN plays a role in Aβ processing, axonal transport and formation of synapses [53]. HSPA5, HSP90AB1are both members of the heat shock protein family and as chaperone proteins are involved in controlling protein folding and ER homeostasis. These candidate biomarkers are interesting and worthy of follow-up to assess possible S2R mechanistic connections, but findings also warrant validation/replication in future trials with zervimesine.

Taken together, our findings from the plasma exploratory proteomic analysis of SHINE participants provide support for a role of zervimesine in impacting amyloid biology, synaptic biology, vesicle and lipid trafficking, neuroinflammation and neurodegeneration (Fig. 6). Alterations in levels of proteins associated with S2R-related functions after 6 months of zervimesine treatment is an encouraging indicator of target/pathway engagement (Fig. 6A). Biomarkers associated with the favorable dampening of GFAP/neuroinflammation were also identified (Fig. 6B). Moreover, comparative analyses with the plasma proteomics from SHINE with proteomics analyses performed from the clinical trial SPARC (COG0105; NCT03493282) identified replication of specific plasma biomarkers, i.e. AHNAK nucleoprotein 2 and ELANE, strengthening the potential relevance of those findings by providing clinical validation of biomarker across cohorts [4].

### Limitations

Limitations of this study include a limited sample size, and sample sizes were even smaller in subgroups. Smaller sample sizes may impact the differential abundance analysis and can lead to identification of false positives or negatives. All proteomics differential abundance and correlations analysis performed considered unadjusted p-values and did not control for multiple comparisons, thus permitting a greater likelihood of type 1 errors (false positives). As a result, candidate biomarkers identified should be viewed with caution until future studies permit replication of these findings. While the p-tau217 subgroup analysis was prespecified, it was not the primary analysis. Moreover, given that plasma was used for the analyses, it is possible that while some biomarkers identified may reflect biological changes in the brain, it is also possible that some biomarkers may reflect changes occurring in the periphery, that may not be disease-relevant. While data may allow for a deeper mechanistic understanding of zervimesine’s biological effects in AD patients, and foster new hypothesis generation, replication of these findings in larger trials and follow-on studies is warranted.

## Conclusion

In summary, for the first time we have evaluated the effect of zervimesine in impacting disease-relevant biomarkers in plasma of patients with AD. In a subgroup analysis of the SHINE trial, in alignment with the clinical effects and CSF biomarker changes, treatment with zervimesine for 6 months leads to positive changes (decreases) in canonical biomarkers of neuroinflammation, trends towards dampening of neurodegeneration, and an impact on amyloid biology; biological effects consistent with the greater clinical effect size seen in the low p-tau217 subgroup. Plasma proteomic analysis of SHINE participant further supports a role of zervimesine in impacting amyloid biology, synaptic biology, vesicle and lipid trafficking, neuroinflammation and neurodegeneration and highlights a newly discovered set of biomarkers to track in future trials, biomarkers which could reflect S2R pathway engagement or an impact on the disease progression. While proteomics analyses were exploratory with limited sample sizes, they provide useful information to support hypothesis generation for future studies to further unravel the mechanistic underpinnings of the biological impact of zervimesine in AD patients. The p-tau217 analysis enabled us to identify a patient population more likely to respond to zervimesine, and may support the enrichment of patients in Phase 3 [22]. In sum, findings of the SHINE study support the continued clinical development of zervimesine for the treatment of neurodegenerative diseases.

## Supplementary Information

Supplementary Material 1.

## Data Availability

The datasets used and/or analyzed during the current study are available from the corresponding author on reasonable request.

## Abbreviations

Aβ: Amyloid beta
AD: Alzheimer’s disease
ADAS-Cog11: Alzheimer’s Disease Assessment Scale – Cognitive Subscale 11-item version
ANOVA: Analysis of variance
CFB: Change from baseline
CSF: Cerebrospinal fluid
DLB: Dementia with Lewy bodies
GO: Gene Ontology
mITT: Modified Intent-to-Treat
PPI: Protein-Protein Interaction
S2R: Sigma-2 receptor, TMEM97
SIMOA: Single molecule array
TMT-MS: Tandem mass tag-mass spectrometry
