# Supplementary material for: Impact of zervimesine on the neuroinflammatory biomarker GFAP and related proteomic molecular correlates in plasma of participants from a phase 2 clinical trial in Alzheimer’s disease
Source: Alzheimers Res Ther. 2026 Apr 6;18:112. doi: 10.1186/s13195-026-02025-4 (PMC13182022; doi:10.1186/s13195-026-02025-4)

**Supplementary Figures and Tables**


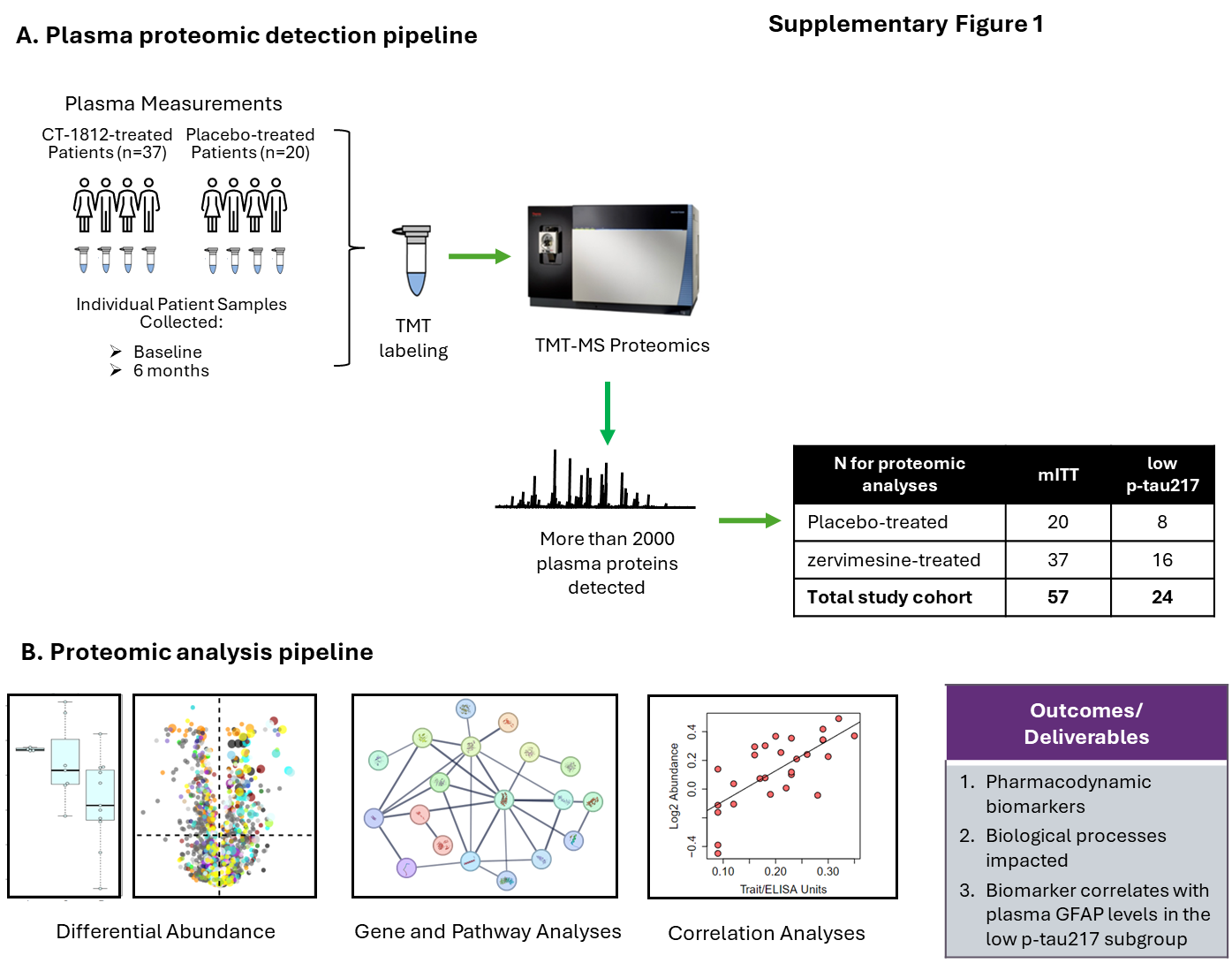


**Supplementary Figure 1. Plasma proteomics detection and analysis pipeline**. **A.** Plasma from the first tranche of participants for whom both baseline and 6 months samples were available and were treatment-compliant were used for differential abundance analyses (N=57 treatment compliant participants, n=20 placebo and n=37 zervimesine). Additional analyses were performed on 24 of the 57 mITT participants which met the criteria for inclusion in the low p-tau217 subgroup (N=24). **B**. Proteome analysis pipeline. Log2 fold change from baseline (CFB) abundances of proteins were calculated and zervimesine vs. placebo significance was calculated by ANOVA for both the mITT and low p-tau217 subgroup. Significantly differentially abundant proteins (zervimesine vs. placebo; p≤0.05) were identified and assessed for interconnectivity and biological relevance using STRING, MetaCore, and Gene Ontology. Pearson correlation analysis was also performed to identify proteomic correlates of plasma GFAP levels.

**Supplementary Table 1**. Baseline characteristics for population with plasma p-tau217 above (high p-tau217 subgroup) or below (low p-tau217 subgroup) the median of 1.0 pg/mL at baseline.

**
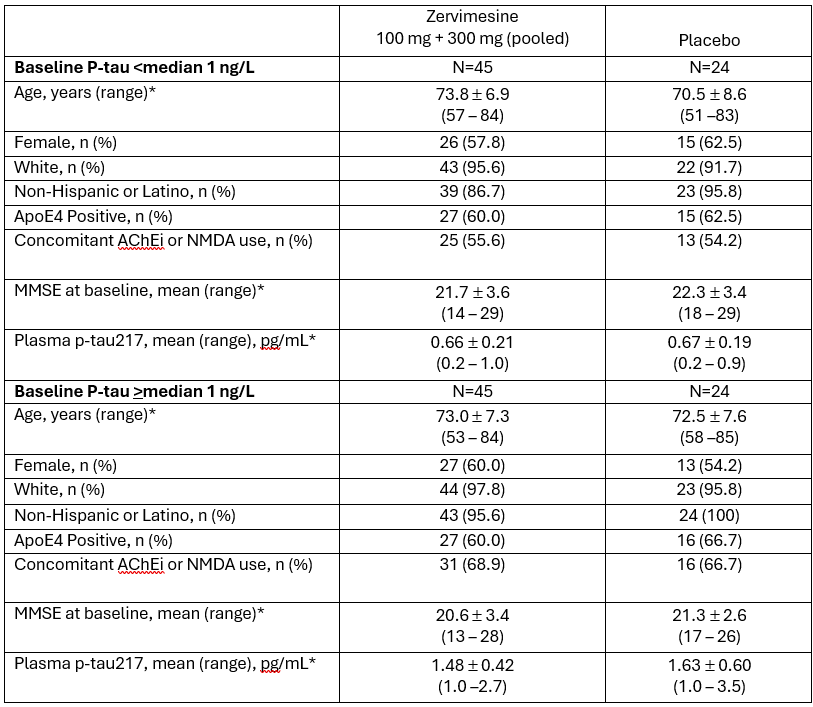
**

**Supplementary Table 2. “Priority AD biomarkers” impacted by 6 months treatment with zervimesine in mITT and low p-tau217 groups**. For these “Priority AD biomarkers”, directionally of change similar across mITT and the low p-tau217 cohorts in the upward direction.


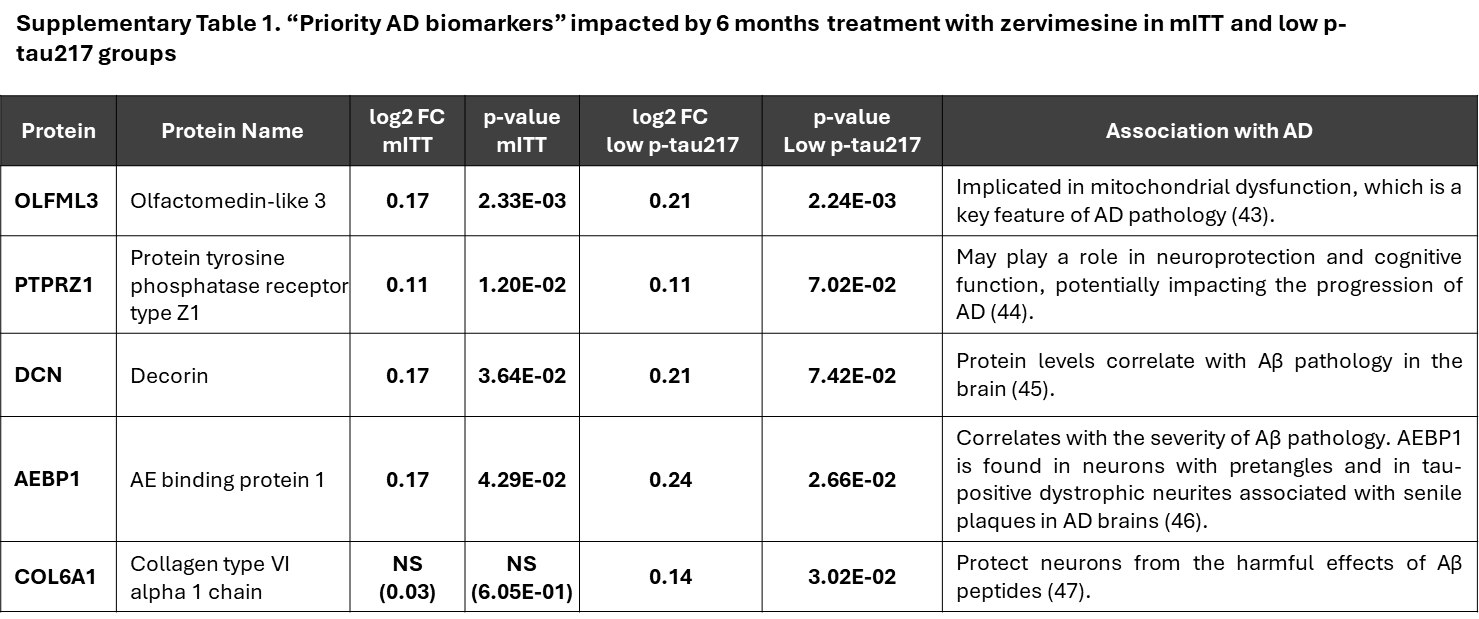


**Supplementary Table 3:** Topmost “Drug Selective” correlates with plasma GFAP in the low p-tau217 group, sorted by correlation r value, with p-value shown.


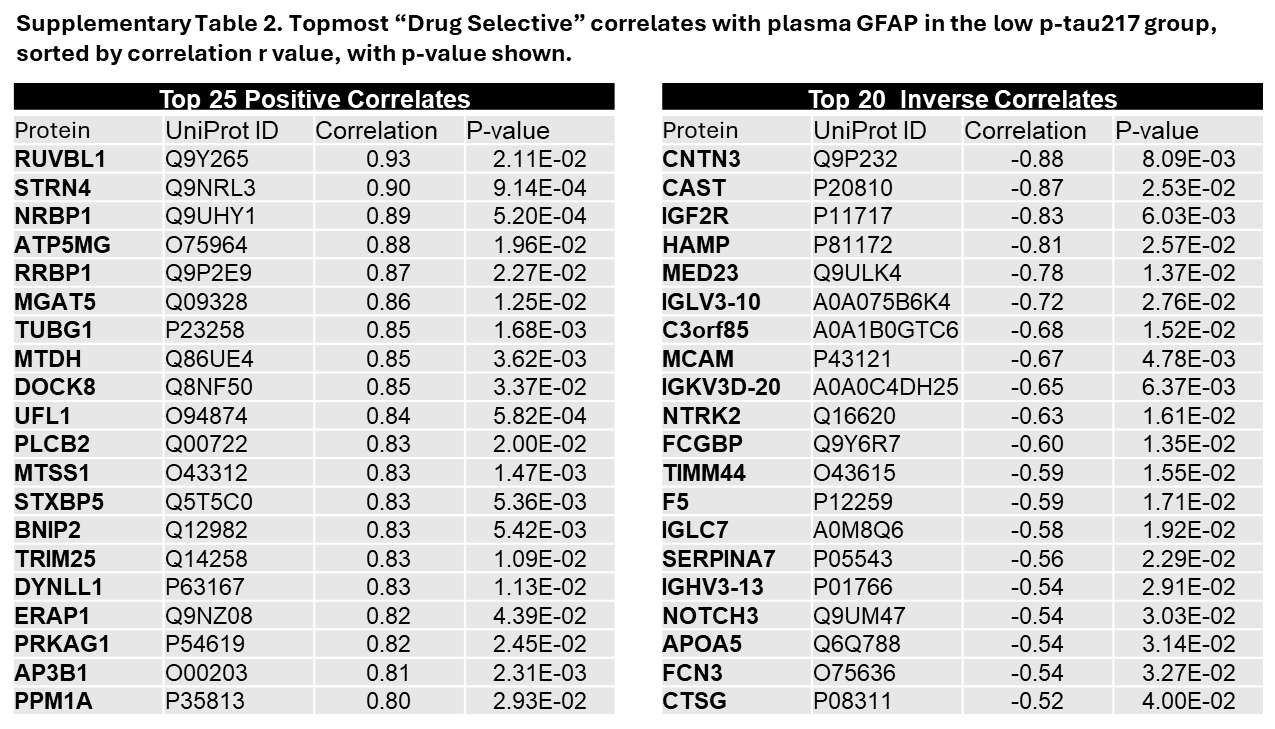

Supplement: Supplementary file 1 — Supplementary Material 1. [file 13195_2026_2025_MOESM1_ESM.docx]
